# CPT1A Alleviates Senescence and Restores Osteogenic Differentiation of BM‐MSC Through SOD2 Succinylation

**DOI:** 10.1111/jcmm.70473

**Published:** 2025-03-11

**Authors:** Xiao Yuan Wang, Shi Chang Liu, Xu Xu Chen, Liang Yan, Liang Li, Gao Le He, Ming Yang, Zhong Kai Liu, Xin Hua Yin

**Affiliations:** ^1^ Physical Examination Center Xi'an International Medical Center Hospital Xi'an China; ^2^ Department of Spine Surgery, Hong Hui Hospital Xi'an Jiaotong University College of Medicine Xi'an China; ^3^ Department of Sports Medicine, Hong Hui Hospital Xi'an Jiaotong University College of Medicine Xi'an China

**Keywords:** BM‐MSC, CPT1A, osteogenic differentiation, SOD2, succinylation

## Abstract

Bone marrow mesenchymal stem cells (BM‐MSCs) have promising prospects in bone repair and regenerative medicine. However, BM‐MSCs gradually lose their original pluripotency and differentiation potential after successive passages. This study aimed to reveal the mechanism underlying the phenomenon. Western blotting, SA‐β‐gal staining and Alizarin red staining were used to evaluate the senescence phenotype and osteogenic differentiation ability. Mitochondrial ROS levels were detected using flow cytometry. Protein interactions and succinylation modifications were identified by using Co‐IP assays. With the increase in passage times, the proliferation and osteogenic differentiation of BM‐MSCs were gradually weakened, and the expression level of CPT1A was decreased. BM‐MSCs with fewer passages (P1–P5 generations) showed increased mitochondrial ROS production and reduced enzyme activity of superoxide dismutase 2 (SOD2) and the mitochondrial level after the knockdown of CPT1A. In contrast, overexpression of CPT1A in multiple‐round‐passed BM‐MSCs cells (P10–P15 generations) has the opposite effect. Therefore, CPT1A level is associated with the ageing phenotypes and the osteogenic differentiation capacity of BM‐MSCs. Knocking down CPT1A significantly reduced the succinylation modification of SOD2, resulting in a decrease in SOD2 enzyme activity and SOD2 levels in mitochondria. Overexpression of CPT1A enhanced the succinylation of SOD2 at the key site K130, thereby reducing cell senescence and promoting osteogenic differentiation. However, this boosting effect was reversed when a mutation occurred at the K130 site of SOD2. CPT1A promotes succinylation modification at the SOD2 (K130) site to induce the accumulation of SOD2 in mitochondria and the enzyme activity, which alleviates BM‐MSC senescence and enhances osteogenic differentiation.

## 1 Introduction

Osteoporosis is a common metabolic disease of bone tissue, characterised by reduced bone mass and increased bone fragility. Mesenchymal stem cells (MSCs) are considered the ideal seed cells for bone repair and regenerative medicine due to their multipotent differentiation potential. Bone marrow mesenchymal stem cells (BM‐MSCs) are a kind of MSC. However, after long‐term culture and continuous passage in vitro, the biological characteristics of MSCs are changed and enter a state of senescence, which limits their potential for clinical applications. Therefore, exploring the mechanisms of MSC senescence and methods to enhance their osteogenic differentiation potential is crucial for bone regenerative medicine.

Mitochondrial superoxide dismutase 2 (SOD2) is a well‐known molecule for scavenging mitochondrial reactive oxygen species (ROS), and it plays a role in preventing cell death, oxidative stress, ionising radiation and inflammatory cytokines [1, 2, 3]. The loss of balance between oxidative and antioxidant systems in cells results in insufficient antioxidant capacity and damage to the cell structure, a state known as ‘oxidative stress’. SOD2, a key reductase in the mitochondrial matrix, is responsible for converting superoxide anions (O^2−^) produced by the electron transport chain into hydrogen peroxide (H_2_O_2_), essential for eliminating intracellular ROS and protecting cells from oxidative stress damage. Studies have found that SOD2 plays a key role in preventing oxidative damage in MSCs. For instance, Sirt3 targets SOD2 to effectively reduce oxidative stress injury in rat BM‐MSCs, thereby delaying the senescence process. Overexpression of SOD2 can protect brain‐derived MSCs from oxidative stress [4]. Furthermore, in the ageing process of umbilical cord blood‐derived MSCs (UCB‐MSCs) induced by TNF‐α, SIRT5 plays a crucial protective role by regulating fatty acid β‐oxidation and SOD2‐mediated antioxidant responses [5]. Despite the importance of SOD2 in MSC senescence and oxidative stress that existing studies have revealed, the specific mechanisms of SOD2 in the ageing process of MSCs still require further investigation.

Increasing evidence suggests that succinylation is involved in the regulation of stem cells. For example, induced succinylation of CDC42 inhibits the proliferation of neural stem cells, thereby exacerbating brain ischaemia/reperfusion injury [6]. Similarly, succinylation elevation mediated by SIRT5 knockdown inhibits the proliferation of adipose‐derived MSCs [7]. lysine acetyltransferase 2A (KAT2A)‐mediated succinylation of notch1 promotes the proliferation and differentiation of dental pulp stem cells through notch pathway activation [8]. Succinylation modification is a newly discovered posttranslational modification (PTM) that can regulate various physiological and pathological processes. Lysine succinylation is an evolutionarily conserved PTM, in which a succinyl group is transferred from succinyl‐CoA to the specific α‐amino of lysine [9]. Notably, KAT2A and carnitine palmitoyltransferase 1A (CPT1A) have recently been identified as succination enzymes [10, 11]. Studies have reported that CPT1A does not only play a significant role in the development of breast cancer [12], gastric cancer [13], ovarian cancer [14], lymphoma [15], vascular smooth muscle cells [10] and pulmonary fibrosis by regulating the succinylation levels of substrate targets [16] but it can also affect the cellular oxidative stress level and antioxidant capacity by regulating mitochondrial function and fatty acid metabolism, thus affecting cellular ageing [17, 18]. However, the role of CPT1A‐related succinylation modifications in MSCs remains poorly understood.

In this study, we focused on BM‐MSCs to investigate the effects of CPT1A on the senescence and osteogenic differentiation of BM‐MSCs and their potential mechanisms. Through public RNA‐Seq database analysis, we found a gradual reduction in the expression level of CPT1A with the passage of BM‐MSCs. Next, we studied the effects of CPT1A knockdown and overexpression on the senescence and osteogenic differentiation of BM‐MSCs and their potential mechanisms. Additionally, we validated the impact of CPT1A on SOD2 succinylation modification by site‐specific mutation of the K130 site in SOD2. This research will provide a new theoretical basis and potential therapeutic strategies for applying BM‐MSCs in bone repair and regenerative medicine.

## 2 Materials and Methods

### 2.1 Cell Culture

The human bone marrow mesenchymal stem cells (BM‐MSCs; catalogue no. PCS‐500‐012) were sourced from the American Type Culture Collection (ATCC) in Manassas, VA, USA, and subsequently cultivated in a low‐glucose Dulbecco's modified Eagle medium (DMEM; D5030, Sigma‐Aldrich, USA). This medium was enhanced with 10% foetal bovine serum (FBS; F2442, Sigma‐Aldrich, USA), along with penicillin at a concentration of 100 U/mL (Invitrogen, USA) and streptomycin at 100 μg/mL (Invitrogen, USA). For the following experiments, BM‐MSCs at Passages 5 and 10 (P5 and P10), which were derived from serial passages, were selected for use.

According to the manufacturer's protocol, BM‐MSCs are induced using osteogenic, lipogenic and chondrogenic differentiation kits (FuHeng Biology; China). 2 × 10^5^ cells of two to three generations were inoculated in 24‐well plates and cultured at 37°C and 5% CO_2_. When the degree of cell fusion reached 70%–80%, the differentiation induction medium was replaced and continued for 2–3 weeks. After culture, the cells were fixed with 4% formaldehyde at room temperature for 30 min, and then bone formation, fat formation and cartilage formation were detected by alizarin red staining, oil red O staining and Alcian blue staining, respectively. In short, cells (1 × 10^4^ cells/cm^2^) were inoculated separately and cultured at 37°C and 5% CO_2_ for 21 days to induce differentiation into adipocytes and osteoblasts. After culture, the cells were fixed with 4% formaldehyde at room temperature for 30 min, oil red O (O1391; Sigma‐Aldrich; Germany) and alizarin red (A5533; Sigma‐Aldrich, Merck KGaA) were stained at room temperature for 2 h and then observed and evaluated under an inverted microscope (Nikon Corp., Japan). BM‐MSCs were incubated in DMEM complete medium at a concentration of 1.6 × 10^7^ cells/ml to induce differentiation into chondrocytes. Subsequently, the StemPro chondrogenic differentiation kit (A1007101; Thermo Fisher Scientific Inc., USA) was inoculated with 8 × 10^4^ cells per well, cultured at 37°C and 5% CO_2_ for 14 days, then fixed with 4% formaldehyde at room temperature for 30 min and stained with Alcian blue (B8438; Sigma‐Aldrich; USA) at room temperature for 2 h.

### 2.2 Flow Cytometry

The immunophenotype of BM‐MSCs was evaluated by flow cytometry, and the expression of mesenchymal stem cell surface markers was analysed using flow cytometry. Briefly, BM‐MSCs were washed with phosphate‐buffered saline (PBS) and incubated in the dark for 15 min at 4°C with the following antibodies: APC‐conjugated anti‐CD73 (clone AD2,560,847), FITC‐conjugated anti‐CD90 (clone 5E10, 561,969), PE‐conjugated anti‐CD105 (clone 266, 560,839) and PerCP‐conjugated anti‐CD34 (clone 8G12) (all from Falcon BD Labware, Franklin Lakes, NJ, USA). After labelling, cells were washed twice, resuspended in PBS and analysed in a BD FACSAria flow cytometer (at least 50,000 cells per condition). The results were analysed using FlowJo software (FlowJo LLC, Ashland, OR, USA).

### 2.3 Cell Transfection

The cells were inoculated in six‐well plates and cultured until 70% confluent. CPT1A gene knockout plasmid (KD‐CPT1A), SOD2 gene knockout plasmid (KD‐SOD2) and control plasmid (KD‐NC) were constructed by General Biological Company (Anhui, China). The CPT1A overexpression vector (CPT1A), SOD2 overexpression vector [SOD2 (WT)], SOD2 overexpressing mutations K122 and K130 [SOD2 (K122R, SOD2) (K130R)] and empty vector were purchased from GenePharma (Shanghai, China). According to the protocol, BM‐MSCs were inoculated in a 6‐well plate and transfected with 100 nM plasmid using Lipofectamine 3000 (Invitrogen, CA, USA). The medium was replaced with a new complete medium 6 h after transfection. After 48 h, the transfected cells were harvested.

KD‐CPT1A or CPT1A(OE) plasmid was transfected into BM‐MSCs cells, and the protein synthesis inhibitor 10 μg/mL actinomycetin cycloheximide (CHX) was added at 0, 2, 4, 8 or 12 h before cell collection to inhibit protein synthesis. The detection time was set to 0, 2, 4, 6 or 10 h, and the cells were collected to detect the expression level of SOD2 compared with internal reference protein.

### 2.4 RNA Isolation and Quantitative Real‐Time Polymerase Chain Reaction (qPCR)

RNA isolation from BM‐MSCs was performed using the Trizol reagent (15,596,026; Invitrogen, USA), and the extracted RNA was stored at 4°C. After quantifying the protein concentration with a NanoDrop 2000 spectrometer (Thermo Fisher Scientific, USA), 1 μg of total RNA was converted into cDNA utilising the SureScript First‐strand cDNA Synthesis Kit (QP057; GeneCopoeia, China). QPCR was carried out with the QuantiTect Reverse Transcription kit (205,314; Hilden, Germany) on a 7500 Fast real‐time PCR System (Thermo Fisher Scientific, USA), employing the following thermal cycling parameters: initial denaturation at 94°C for 10 min, followed by 40 cycles of denaturation at 94°C for 30 s, annealing at 60°C for 30 s and extension at 72°C for 40 s. The primer sequences are detailed in Table 1, with GAPDH and U6 serving as internal controls. The gene expression levels were determined using the 2 − ΔΔCT method.

**TABLE 1:** Primer sequences.

Primer	Forward (5′ → 3′)	Reverse (5′ → 3′)
CPT1A	TCCAGTTGGCTTATCGTGGTG	TCCAGAGTCCGATTGATTTTTGC
SOD2	GGAAGCCATCAAACGTGACTT	CCCGTTCCTTATTGAAACCAAGC
β‐Actin	CATTAAGGAGAAGCTGTGCT	GTTGAAGGTAGTTTCGTGGA

### 2.5 Western Blotting and Coimmunoprecipitation (Co‐IP)

The BM‐MSCs cells had their proteins extracted using RIPA reagent (Thermo Fisher Scientific, USA). The total protein concentration was measured using a BCA Protein Assay Kit (Beyotime, China). Next, 20 μg of protein was subjected to separation via 10% SDS‐PAGE and subsequently transferred onto a PVDF membrane (Merck, China). The membranes underwent blocking with 5% skim milk for 1 h before being exposed to primary antibodies overnight at 4°C. After that, HRP‐conjugated secondary antibodies were applied to the membrane for a 1‐h incubation. Protein visualisation was achieved using the ImageQuant LAS‐4000 mini (GE Healthcare, USA), and the relative protein levels were quantified with ImageJ software. Co‐IP was conducted to validate the endogenous interaction between CPT1A and SOD2 succinylation. Anti‐CPT1A, antisuccinyllysin and normal IgG were introduced to 1 mg of cell lysates that had been precleared with protein G agarose beads, followed by an overnight incubation at 4°C. The co‐IP complexes were then collected and subjected to western blot analysis. To perform CPT1A‐related protein silver staining and mass spectrometry analysis, BM‐MSC cells were transfected with FLAG‐CPT1A and FLAG‐NC control plasmids. Cells were collected and lysed (including protease inhibitors and phosphatase inhibitors) 48 h after transfection. Immunoprecipitation was performed using anti‐Flag antibody (Abcam, United Kingdom), followed by protein mass spectrometry analysis and silver staining of the protein gel was conducted on total cellular protein (Input), IgG elution samples and IP elution samples. Details of all antibodies employed in this study are provided in Table 2.

**TABLE 2:** Information of antibodies.

Primary antibodies	MW (kDa)	Dilution	Company/catalogue	Secondary antibodies	Dilution
Succinyllysine	/	1:500	PTM, PTM‐401	Goat anti‐rabbit IgG/HRP	1:4000
SOD2	≈23	1:2000	Ptgcn, 24,127‐1‐AP	Goat anti‐rabbit IgG/HRP	1:4000
CPT1A	≈86	1:1000	Ptgcn, 66,039‐1‐Ig	Goat anti‐mouse IgG/HRP	1:4000
Runx2	≈57	1:1000	Abcam, ab76956	Goat anti‐mouse IgG/HRP	1:4000
Osteocalcin	≈11	1:400	Abcam, ab93876	Goat anti‐rabbit IgG/HRP	1:4000
p16	≈16	1:500	Ptgcn,10,883‐1‐AP	Goat anti‐rabbit IgG/HRP	1:4000
p21	≈21	1:500	Ptgcn, 10,355‐1‐AP	Goat anti‐rabbit IgG/HRP	1:4000
HA		1:2000	Ptgcn,51,064‐2‐AP	Goat anti‐rabbit IgG/HRP	1:4000
FLAG		1:800	Abcam, ab205606	Goat anti‐rabbit IgG/HRP	1:4000
β‐Actin	42	1:2000	Ptgcn, 66,009‐1‐Ig	Goat anti‐mouse IgG/HRP	1:4000

### 2.6 Senescence‐Associated β‐Galactosidase (SA‐β‐Gal) Activity Assay

The BM‐MSCs cultivated in 12‐well plates were subjected to fixation and staining utilising a senescence cells histochemical staining kit, following the protocol provided by the manufacturer. The fixed cells were treated with the staining solution mixture and incubated in a dry incubator, free of CO_2_, at 37°C for 12 h. The proportion of cells displaying a positive stain (appearing blue) was determined for each experimental group by examining five randomly chosen fields under a 100× magnification bright‐field microscope.

### 2.7 Immunofluorescence Colocalisation

BM‐MSCs at a 1 × 10^5^/mL concentration were plated in a 35 mm cell culture dish featuring a glass bottom. After allowing the cells to adhere, they were fixed with 4% paraformaldehyde for 15 min and then permeabilised using PBS with 0.3% TritonX‐100 for an additional 15 min. The cells were subsequently blocked with 5% FBS in PBS for 1 h before exposure to anti‐SOD2 and MitoTracker Green dye overnight at 4°C. A 2‐h incubation with an Alexa Fluor 647‐conjugated goat anti‐Rabbit IgG secondary antibody followed this. Finally, the BM‐MSCs were mounted with DAPI and fluorescence images were acquired with a light microscope (Leica DM 2500, Germany) at 400× magnification.

### 2.8 Measurement of SOD2 Enzyme Activity

SOD2 enzymatic activity was assayed using a superoxide dismutase (Mn‐SOD) assay kit with WST‐1 (Nanjing Jiancheng, China). Following trypsinisation, the MSCs were adjusted to a concentration of 1 × 10^6^ cells per millilitre. These cells were then subjected to two washes with PBS and centrifuged at a speed of 1000 rpm for 10 min, after which the supernatant was discarded. The resultant pellet from the centrifugation was lysed using an ultrasonic process, and the lysates were subsequently resuspended. The SOD2 activity was assessed by the manufacturer's guidelines, utilising a microplate reader.

### 2.9 Mitochondrial ROS Level Detection (MitoSOX)

Mitochondrial superoxide levels in UCB‐MSCs were quantified using a flow cytometer (Cell Lab Quanta SC, USA) through the following procedure: After trypsinisation, the UCB‐MSCs were treated with 5 μM MitoSOX and incubated for 20 min at 37°C, ensuring the solution was shielded from light. A single wash with PBS was performed before the cells were resuspended in PBS for subsequent analysis via flow cytometry. The data obtained were processed to determine the percentage of cells exhibiting high fluorescence intensity (red) with Flowing software 2 (developed by Perttu Terho, Turku, Finland).

### 2.10 Liquid Chromatography–Tandem Mass Spectrometry Analysis (LC–MS)

First, the sample was ultrasonically lysed with lysis buffer (8 M urea, 1% protease inhibitor, 3 μM TSA and 50 mM NAM), and the protein concentration was determined by the BCA method. The same amount of protein was precipitated by TCA, enzymolysed by trypsin, then reduced and alkylated by DTT and IAM. The peptides are then combined with the succinylated resin, incubated overnight and washed before being desalted according to the C18 ZipTips instructions. Finally, the peptides were separated in a liquid chromatography system and detected at high resolution by a timsTOF Pro mass spectrometer. The PASEF model was used to collect data, ensuring the identification of modification sites accurately.

### 2.11 Statistical Analysis

Each experimental procedure was conducted a minimum of three times to ensure reliability. The error bars presented represent the ± standard deviation (SD). All data were in a normal distribution, and variance was similar between the groups being statistically compared. Statistical analyses were analysed in GraphPad Prism 8. Statistical significance was determined by using the unpaired Student t‐test for two groups or one‐way ANOVA when there are more than two groups. *p* < 0.05 was considered significant.

## 3 Result

### 3.1 A Gradual Reduction of the Expression Level of CPT1A With the Passage of BM‐MSCs

**FIGURE 1:**
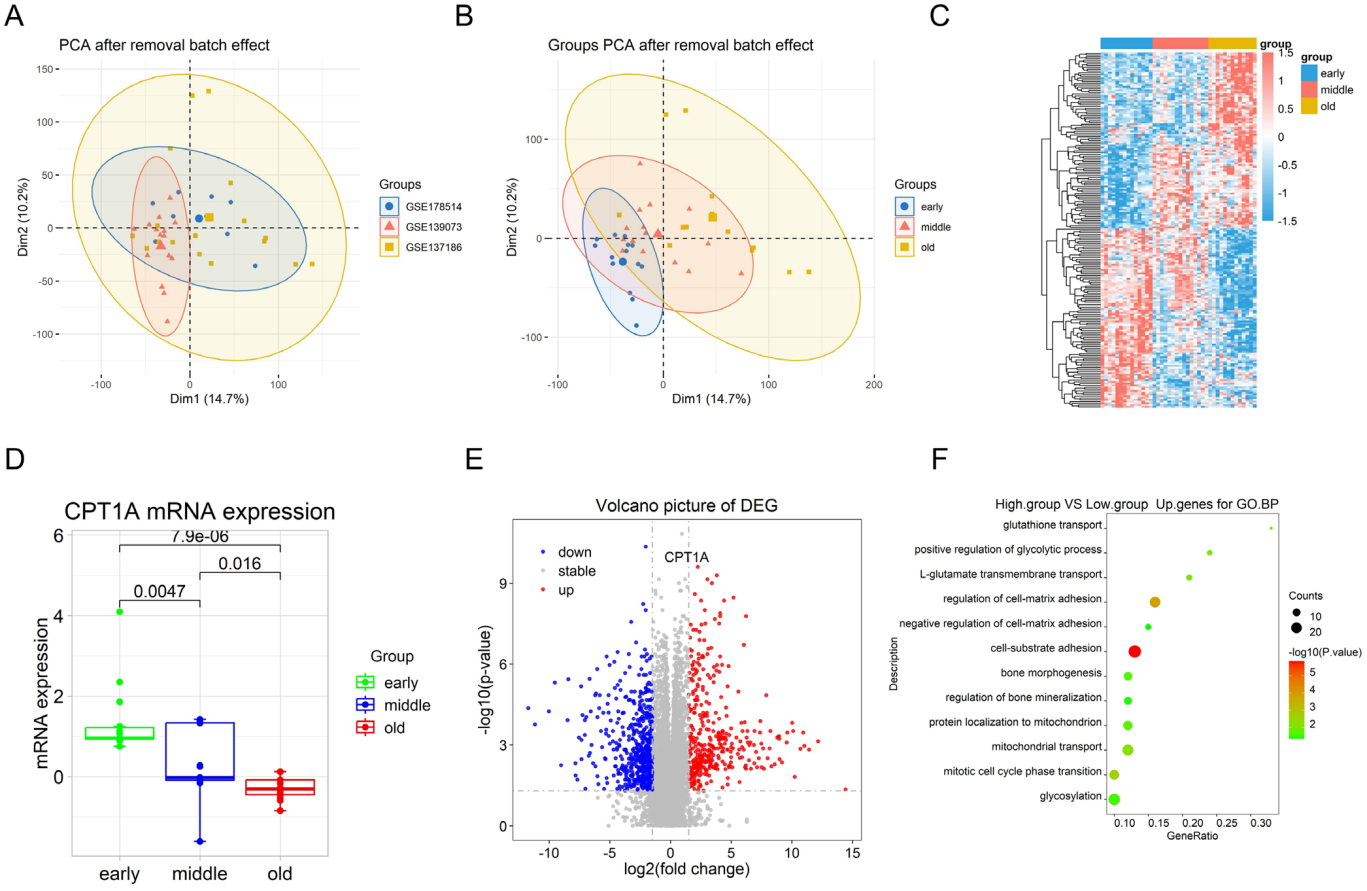
CPT1A was negatively correlated with the passage times of BM‐MSCs. (A) Sva packages in R language to remove batch effects. (B) Grouping samples after batch removal. (C) Heatmaps of the first 200 differential genes DEGs (100 up and 100 down). (D) CPT1A gene expression differences among the three groups. (E) DEGs volcano map of differentiated genes after CPT1A‐up and CPT1A‐down grouping. (F) GO enrichment analysis of CPT1A‐down group genes (*n* = 21 for each group).

First, we used three different public RNA‐Seq databases (GSE139073, GSE178514 and GSE137186) to analyse changes in the gene expression of BM‐MSCs at different passage times. After using the sva package in R language to remove batch effect, the samples were divided into three groups: early passage (1–3 generations), middle passage (4–6 generations) and late passage (≥ 10 generations) (Figure 1A,B). Then, the differentially expressed genes (DEGs) selected by the limma formula were used, and the first 200 (100 positive and negative) were selected for subsequent analysis and heatmaps were drawn (Figure 1C). CPT1A expression was gradually reduced with the passage times (Figure 1D). To determine the gene enrichment associated with the downregulation of CPT1A gene expression, the samples were divided into CPT1A‐up (upregulated expression group) and CPT1A‐down (downregulated expression group), and the differentially expressed genes (DEGs) between the samples of the two groups were analysed and gene set enrichment analysis (GO analysis) was performed (Figure 1E,F). The results showed that CPT1A was associated with oxidative stress, cell senescence, osteogenic differentiation and other pathways. In particular, the CPT1A‐down gene set showed cellular stress senescence and replicative senescence, negative regulation of osteogenic differentiation and enrichment of cellular oxidative stress pathway genes. In addition, gene enrichment of pathways such as mitochondrial protein targeting, mitochondrial autophagy regulation and intracellular protein stability regulation was also found. Therefore, we chose CPT1A for further investigation.

**FIGURE 2:**
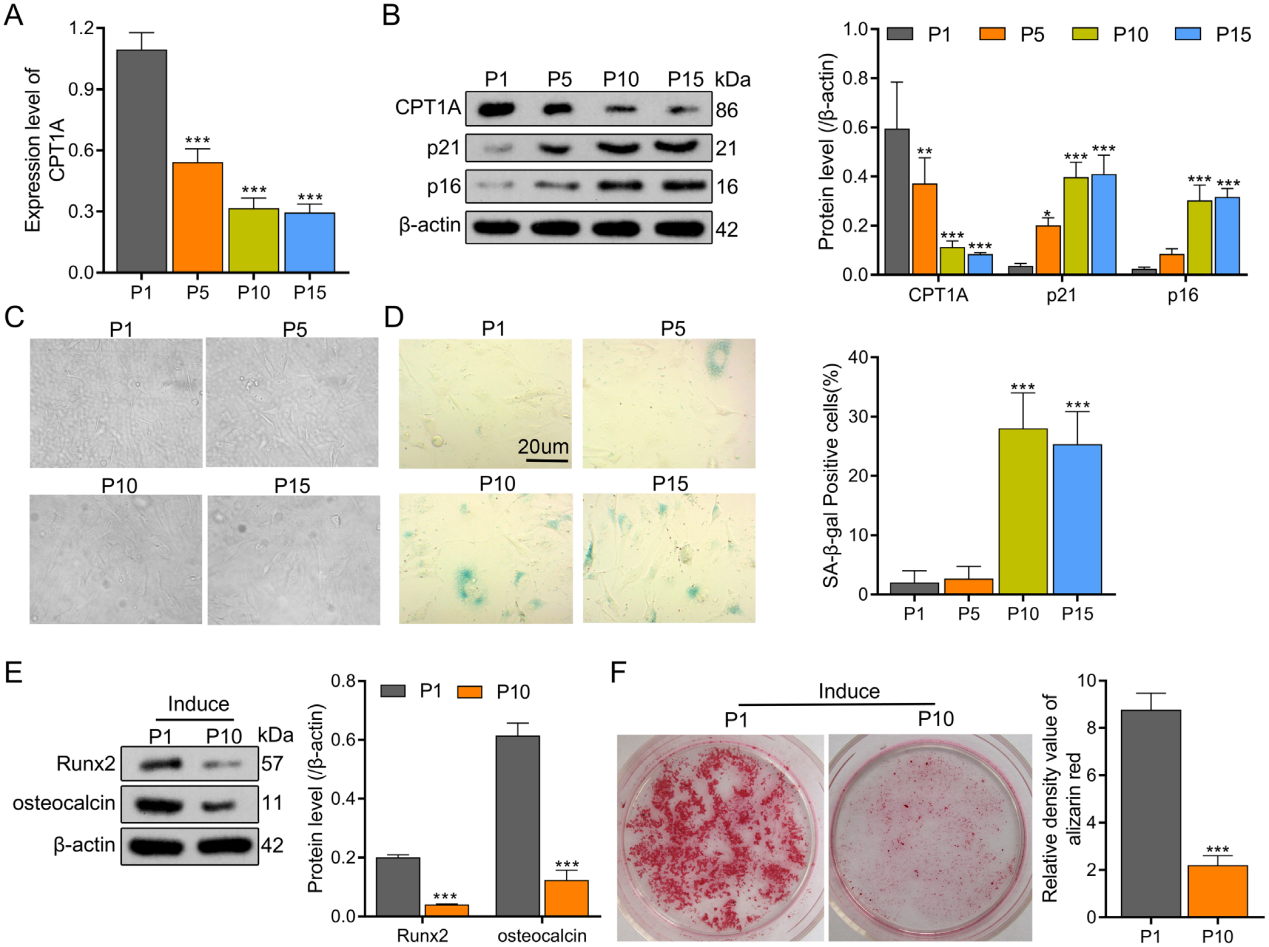
Downregulation of CPT1A expression in BM‐MSCs accelerates senescence and impairs osteogenic differentiation ability during in vitro continuous passaging. (A) The expression of CPT1A mRNA in BM‐MSCs cells with different passage times was detected by qPCR. (B) The protein expression levels of CPT1A and senescence‐related factors p21 and p16 were detected by western blotting. (C) The morphological changes of cells were observed by white light photography. (D) Senescence‐associated SA‐β‐gal‐staining and quantitative analysis (E) The expression levels of Runx2 and osteocalcin were detected by western blotting. (F) Alizarin red staining evaluated the osteogenic differentiation ability of cells. *p < 0.05, **p < 0.01, ***p < 0.001 versus P1. *N* = 3, Statistical significance was determined by using unpaired Student t‐test for two groups or one‐way ANOVA when there are more than two groups. *p* < 0.05 was considered significant.

To identify BM‐MSCs, firstly, by detecting the cell surface markers of MSC, it was found that the positive rate of CD105, CD73 and CD90 was ≥ 95%, and the positive rate of CD34 was ≤ 5% (Figure S1A). Then, after induction of osteogenic, lipogenic and chondrogenic differentiation, BM‐MSCs could successfully differentiate into osteoblasts, adipocytes and chondrocytes, indicating that MSC could differentiate, and the cells were identified as BM‐MSCs (Figure S1B–D). To explore the correlation between the senescence phenotype and osteogenic differentiation capacity of BM‐MSCs cells and the expression of CPT1A during continuous passage, we conducted qPCR and Western blotting analyses on BM‐MSCs cells at Passages 1, 5, 10 and 15. We observed that the expression level of CPT1A gradually decreased with increasing passages (Figure 2A,B), while the levels of senescence‐related proteins p21 and p16 gradually increased (Figure 2B). Additionally, in cells at Passages 10–15, the senescence phenotype was particularly pronounced, characterised by flattened and irregular cell morphology, reduced cell aspect ratio and increased single cell area, significantly different from those of Passage 1 cells (Figure 2C). SA‐β‐gal staining, a classical standard for assessing cellular senescence (Dimri et al., 1995), revealed a significant increase in SA‐β‐gal‐positive blue cells, indicating enhanced enzyme activity associated with senescence (Figure 2D). To assess the osteogenic differentiation capacity of BM‐MSCs cells, we differentiated cells with high (P1) and low (P10) CPT1A expression for 10 days. The expression levels of osteogenic differentiation markers Runx2 and osteocalcin in P1 cells were significantly higher than in P10 cells (Figure 2E). Alizarin red staining further confirmed that the osteogenic differentiation capacity of P10 cells was markedly weaker than that of P1 cells, with reduced formation of calcium nodules (Figure 2F). In summary, during the continuous passage of BM‐MSCs in vitro, the expression of CPT1A was downregulated and the ageing phenotype was gradually obvious, accompanied by the weakening of osteogenic differentiation ability.

### 3.2 CPT1A Enhances SOD2 Enzyme Activity and Mitochondrial Localisation to Promote Osteogenic Differentiation in BM‐MSCs

To explore the effects of CPT1A on the senescence and osteogenic differentiation of BM‐MSCs, we induced osteogenic differentiation in BM‐MSCs cells and transfected P1 BM‐MSCs cells with a CPT1A knockdown plasmid (CPT1A(KD)) as well as P10 BM‐MSCs cells with a CPT1A overexpression plasmid (CPT1A). In P1 BM‐MSCs cells, the expression levels of senescence‐related proteins p21 and p16 did not significantly change compared to the control group after the osteogenic induction, while the expression levels of osteogenic differentiation‐related proteins Runx2 and osteocalcin significantly increased. After knocking down CPT1A, the expression levels of p21 and p16 proteins significantly increased, while the expression levels of Runx2 and osteocalcin proteins significantly decreased. These results indicate that knocking down CPT1A may inhibit the osteogenic differentiation capacity of BM‐MSCs cells. In P10 BM‐MSCs cells, the expression levels of p21 and p16 did not significantly change compared to the control group after induction, while Runx2 and osteocalcin expression increased. After overexpressing CPT1A, the expression levels of p21 and p16 proteins significantly decreased, while Runx2 and osteocalcin expression increased, suggesting that CPT1A overexpression can reinduce osteogenic differentiation in senescent cells (A).

**FIGURE 3:**
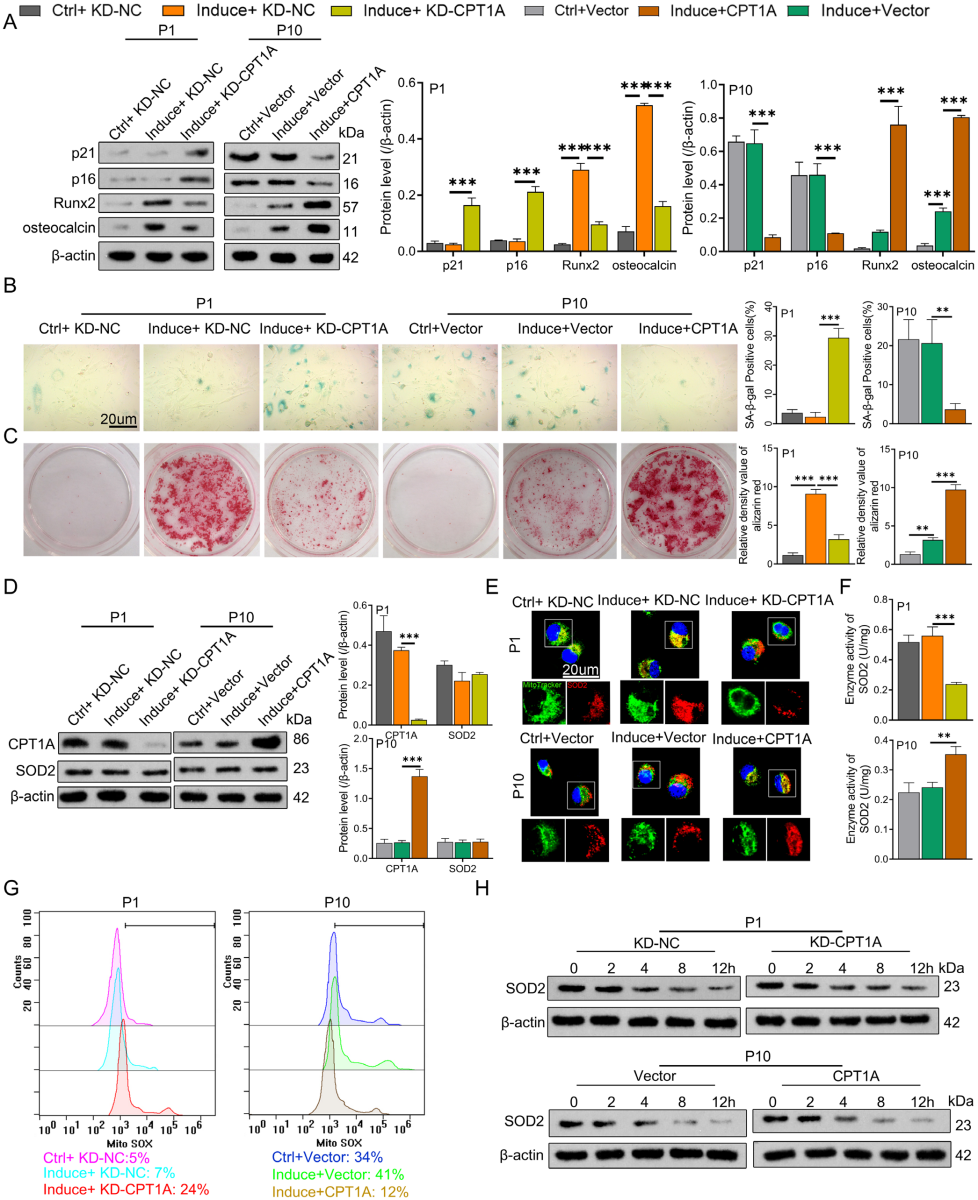
CPT1A enhances SOD2 enzyme activity and mitochondrial localisation to promote osteogenic differentiation in BM‐MSCs. (A) After osteogenic induction culture, CPT1A(KD) or CPT1A plasmid was transfected into BM‐MSCs, and the expression levels of senescence‐related proteins p21 and p16 and osteogenic differentiation‐related proteins Runx2 and osteocalcin were detected by western blotting. (B) SA‐β‐gal staining and quantitative analysis. (C) Alizarin red staining was used to evaluate the osteogenic differentiation of cells. (D) The protein expression levels of CPT1A and SOD2 were detected by western blotting. (E) Colocalisation images of SOD2 and mitochondria were obtained by immunofluorescence. (F) SOD activity detection kit (WST‐8 method) was used to detect SOD activity. (G) Representative images of MitoSox in cells were analysed by flow cytometry to assess mitochondrial ROS levels. (H) CPT1A(KD) or CPT1A plasmid was transfected into BM‐MSCs cells, and the protein synthesis inhibitor 10 μg/mL actinone cycloheximide (CHX) was added 0, 2, 4, 8 and 12 h before cell collection to inhibit protein synthesis. Western blotting detected the protein expression levels of SOD2 at 0, 2, 4, 6 and 10 h. **p < 0.01, ***p < 0.001. *N* = 3, Statistical significance was determined by using one‐way ANOVA. *p* < 0.05 was considered significant.

Additionally, SA‐β‐gal‐staining results showed that the number of SA‐β‐gal‐positive blue cells did not significantly change in P1 cells after osteogenic induction. Still, the number of SA‐β‐gal‐positive blue cells increased significantly after CPT1A knockout. In contrast, the number of SA‐β‐gal‐positive blue cells decreased substantially after CPT1A overexpression in P10 cells (Figure 3B). Alizarin red staining showed that in P1 cells, the degree of alizarin red staining was significantly enhanced in the induction group. In contrast, the degree of alizarin red staining was significantly weakened after CPT1A knockdown. It was also verified in P10 cells that compared with the induction group, the degree of alizarin red staining of cells overexpressing CPT1A after induction differentiation was enhanced (Figure 3C). In summary, knocking down CPT1A may inhibit the osteogenic differentiation capacity of BM‐MSCs cells and promote cellular senescence, while CPT1A overexpression can reinduce osteogenic differentiation in senescent cells and reduce cellular senescence. These results indicate that the expression level of CPT1A is closely related to the senescence and osteogenic differentiation capacity of BM‐MSCs cells.

Recent studies have demonstrated that SOD2 plays a role in the resistance to oxidative damage in the antioxidant therapy of MSC [4]. In addition, CPT1A (OE) plasmid was overexpressed in long‐term cultured MSC cells in vitro, and NC vector was used as the control transfection group for the modification of lysine K succinylation protein. Analysis of the results of silver staining and LC–MS showed that SOD was one of the CPT1A‐interacting proteins when the confidence set was 95% and unique peptides ≥ 1 (Figure S2). SOD2‐K succinylation modification identification and analysis are described below. Therefore, we speculated that SOD2 might be involved in the ageing and osteogenic differentiation process of BM‐MSCs regulated by CPT1A by influencing the antioxidant capacity of BM‐MSCs. Subsequently, we detected the expression changes of CPT1A and SOD2 proteins during osteogenic induction in P1 and P10 BM‐MSCs cells. The results showed that the expression of CPT1A and SOD2 proteins did not significantly change in P1 cells after osteogenic induction. However, after knocking down CPT1A expression, CPT1A protein expression significantly decreased, but SOD2 expression did not change significantly. Similarly, in P10 cells, there were no significant changes in CPT1A and SOD2 protein expression after osteogenic induction. After overexpressing CPT1A, CPT1A protein expression significantly increased, while SOD2 expression remained unchanged (Figure 3D). Immunofluorescence results further indicated that after induction in P1 or P10 cells, the expression of SOD2 in mitochondria did not change significantly, while after knocking down CPT1A or overexpressing CPT1A, the expression of SOD2 in mitochondria significantly decreased or increased (Figure 3E). Moreover, we measured the enzyme activity of SOD2, and the results showed that the enzyme activity of SOD2 did not significantly change after induction in P1 or P10 cells, while after knocking down CPT1A expression or overexpressing CPT1A, the enzyme activity of SOD2 significantly decreased or increased (Figure 3F). This suggests that CPT1A can affect the mitochondrial localisation and enzyme activity of SOD2 and may also regulate antioxidant function. Therefore, we detected the levels of reactive oxygen species (ROS) in mitochondria using the mitochondrial ROS assay and found that after induction in P1 or P10 cells, the mitochondrial ROS levels did not significantly change, while after knocking down CPT1A expression or overexpressing CPT1A, the mitochondrial ROS levels significantly increased or decreased (Figure 3G). These results indicate that CPT1A can promote the mitochondrial localisation of SOD2 and enhance its enzyme activity and antioxidant function. To further investigate whether CPT1A affects the stability of the SOD2 protein, we treated BM‐MSCs cells with the protein synthesis inhibitor cycloheximide. The results showed that knocking down or overexpressing CPT1A in P1 and P10 cells did not significantly affect the degradation of SOD2, indicating that CPT1A does not affect the protein stability of SOD2 (Figure 3H). In summary, our study demonstrates that CPT1A influences the senescence and osteogenic differentiation of BM‐MSCs cells by promoting mitochondrial localisation and enhancing the enzyme activity of SOD2.

### 3.3 CPT1A‐Mediated SOD2 Succinylation and its Effect on Senescence and Osteogenic Differentiation of BM‐MSCs

**FIGURE 4:**
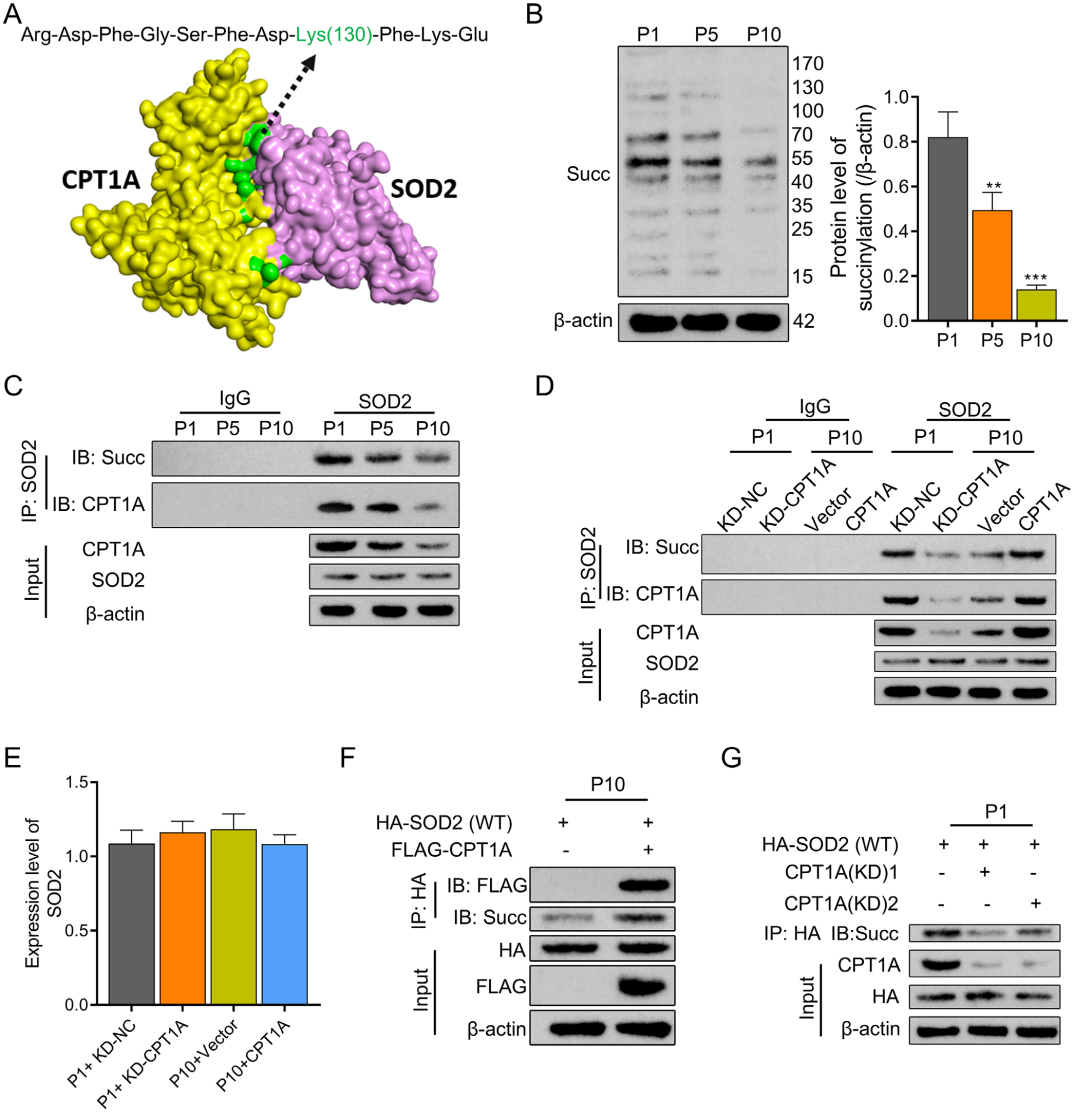
CPT1A‐mediated SOD2 succinylation and its effect on senescence and osteogenic differentiation of BM‐MSCs. (A) Using HDOCK protein on the server (http://hdock.phys.hust.edu.cn)—protein docking analysis tools SOD2 and CPT1A interaction model and through the Discovery of Visual Studio 4.5 software. (B) The global succinylation level of M‐MSCs in Passages 5 and 10 was detected by Western blotting. (C) Co‐IP detected the binding of SOD2 to succinylation and CPT1A. (D) Transfected CPT1A(KD) or CPT1A plasmid into BM‐MSCs, and Co‐IP detected SOD2 binding to succinylation and CPT1A. (E) The expression of SOD2 mRNA was detected by RT‐qPCR. (F) FLAG‐CPT1A and HA‐SOD2 (WT) were constructed and transfected into the 10th generation BM‐MSCs cells, and FLAG (CPT1A) and SOD2‐succinylation were detected by HA antibody IP and IB. (G) BM‐MSCs (the first generation of cells) were transfected with CPT1A (KD) and HA‐SOD2 (WT) tag plasmids, and after HA antibody IP and IB were used to detect HA and SOD2 succinylation. **p < 0.01, ***p < 0.001. *N* = 3, Statistical significance was determined by using one‐way ANOVA. *p* < 0.05 was considered significant.

Using the HDOCK server (http://hdock.phys.hust.edu.cn), the interaction between the SOD2 and CPT1A model is analysed. As shown by the Discovery Studio 4.5 software visualisation, the green ball represents the amino acids (RDFGSFDKFKE) in SOD2 recognised by CPT1A. In the amino acids, SOD2‐Lys(130) is a succinylation site (Figure 4A). To investigate whether CPT1A mediates the succinylation modification of SOD2, we measured the succinylation levels in BM‐MSCs cells at P1, P5 and P10. We found that with increasing passages, the overall succinylation level in the cells was significantly downregulated (Figure 4B). Additionally, through Co‐IP experiments, we observed that in P1 cells, the interaction between SOD2 and CPT1A, as well as SOD2 succinylation modification, was evident, whereas in P10 cells, this interaction and modification were significantly weakened (Figure 4C). These results suggest that CPT1A may be involved in the succinylation modification process of SOD2. To validate the effect of CPT1A on SOD2 succinylation modification, we knocked down or overexpressed CPT1A in BM‐MSCs cells from P1 and P10, respectively. The results showed that under conditions of CPT1A knockdown, the succinylation modification of SOD2 was weakened, whereas overexpression of CPT1A enhanced this modification (Figure 4D), while the mRNA expression level of SOD did not show significant changes (Figure 4E). These results further support the hypothesis that CPT1A mediates the succinylation modification of SOD2.

Next, we verified the interaction between CPT1A and SOD2 in BM‐MSCs with low CPT1A expression. Through exogenous Co‐IP experiments, we found that the transfection of exogenous FLAG‐CPT1A enhanced the interaction with HA‐SOD2 and succinylation modification (Figure 4F). Similarly, in BM‐MSCs with high CPT1A expression, knocking down CPT1A significantly reduced the succinylation level of HA‐SOD2 (WT), further confirming the promoting effect of CPT1A on the succinylation modification of SOD2 in BM‐MSCs (Figure 4G).

### 3.4 ST1326 Promotes the Senescence of BM‐MSCs and Inhibits Osteogenic Differentiation by Reducing CPT1A‐Mediated SOD2 Succinylation

**FIGURE 5:**
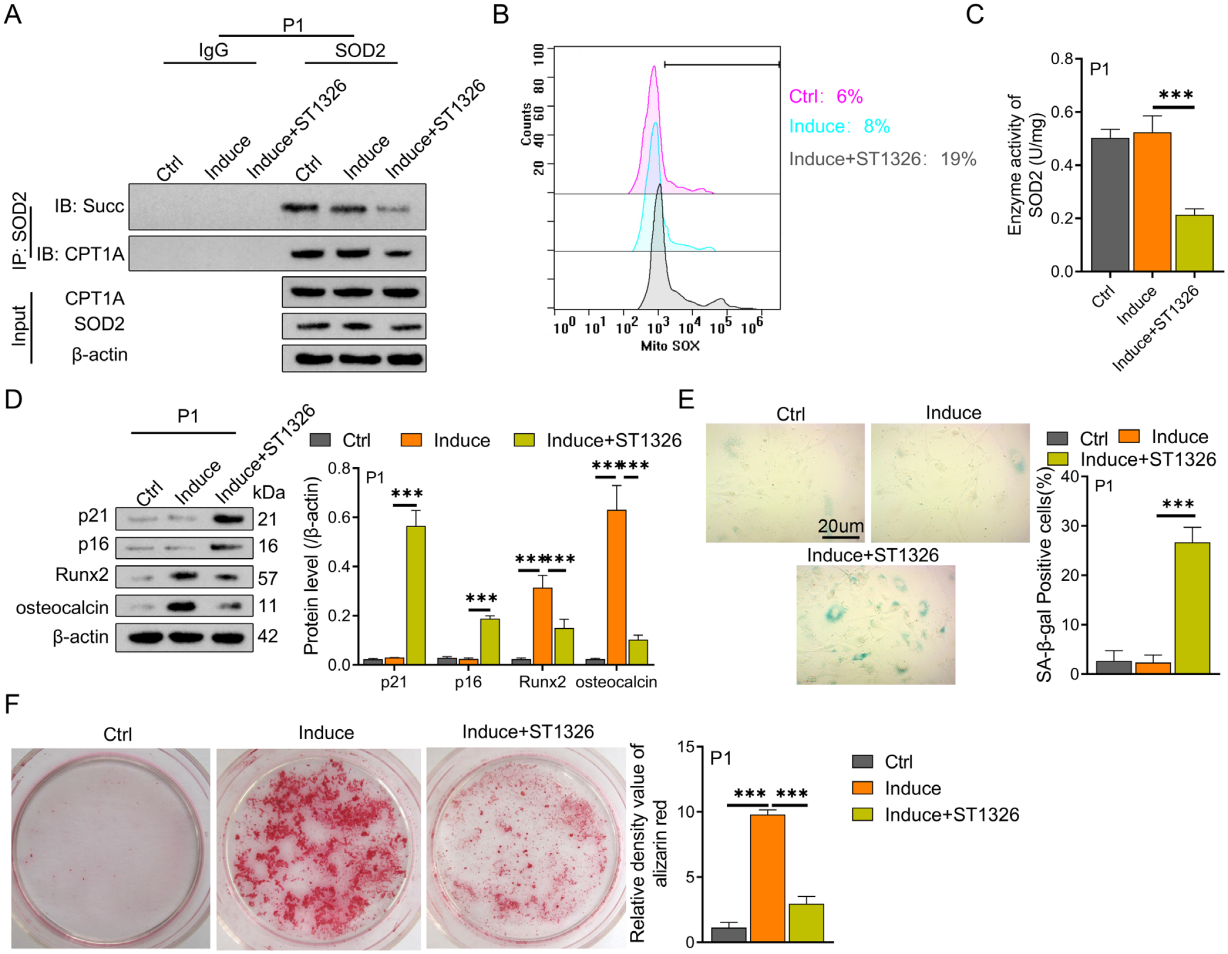
ST1326 promotes senescence of BM‐MSCs and inhibits osteogenic differentiation by reducing CPT1A‐mediated SOD2 succinylation. (A) ST1326‐treated–induced cells Co‐IP detects the combination of CPT1A and SOD2. (B) Representative images of MitoSox in cells were analysed by flow cytometry to assess mitochondrial ROS levels. (C) SOD activity detection kit (WST‐8 method) was used to detect SOD activity. (D) The protein expression levels of p21, p16, Runx2 and osteocalcin were detected by western blotting. (E) SA‐β‐gal staining and quantification of the number of SA‐β‐gal‐positive cells. (F) Alizarin red staining evaluated the osteogenic differentiation ability of cells. ***p < 0.001. *N* = 3, Statistical significance was determined by using one‐way ANOVA. *p* < 0.05 was considered significant.

Subsequently, we further investigated the effects of CPT1A on SOD2 activity and BM‐MSCs cell osteogenic differentiation. Teglicar (ST1326) is a selective and reversible carnitine palmitoyltransferase 1 (L‐CPT1) liver isoform inhibitor with the ability to inhibit CPT1A function. We inhibited the expression of CPT1A in P1 BM‐MSCs cells by treating the cells with 10 μM CPT1A inhibitor ST1326 for 48 h [19, 20]. Co‐IP experiment results showed that after osteogenic induction, there was no significant change in the interaction between SOD2 and succinylation level with CPT1A compared to the control group. However, after treatment with ST1326, the succinylation level of SOD2 and its interaction with CPT1A decreased, while the total levels of SOD2 and CPT1A protein remained unchanged. This indicates that ST1326 inhibited the succinylation process of SOD2 by inhibiting the activity of CPT1A but did not affect the total amounts of SOD2 and CPT1A (Figure 5A). Moreover, after treatment with ST1326, the mitochondrial ROS level significantly increased (Figure 5B) and the enzyme activity of SOD2 decreased (Figure 5C). We detected the protein levels of p21, p16 and osteogenic differentiation‐related markers Runx2 and osteocalcin through Western blotting. We found that compared to the induced group, after treatment with ST1326, the protein expression levels of p21 and p16 significantly increased, while the expression levels of Runx2 and osteocalcin decreased (Figure 5D). SA‐β‐gal‐staining results showed that after treatment with ST1326, the number of senescence‐related SA‐β‐gal‐positive blue cells increased, suggesting that ST1326 may promote the senescence process of the cells (Figure 5E). Alizarin red‐staining results showed that after treatment with ST1326, the intensity of alizarin red staining significantly decreased, further indicating that ST1326 significantly inhibited the osteogenic differentiation ability of BM‐MSCs cells by inhibiting the activity of CPT1A (Figure 5F). In summary, our study results suggest that ST1326 inhibits the succinylation process of SOD2 by inhibiting the activity of CPT1A, thereby reducing the antioxidant function and enzyme activity of SOD2. Additionally, ST1326 may promote the senescence process of the cells and significantly inhibit the osteogenic differentiation ability of BM‐MSCs cells.

### 3.5 The Role of CPT1A in Reducing Senescence and Restoring Osteogenic Differentiation in Expanded BM‐MSCs by Modifying SOD2 (K130) Succinylation

**FIGURE 6:**
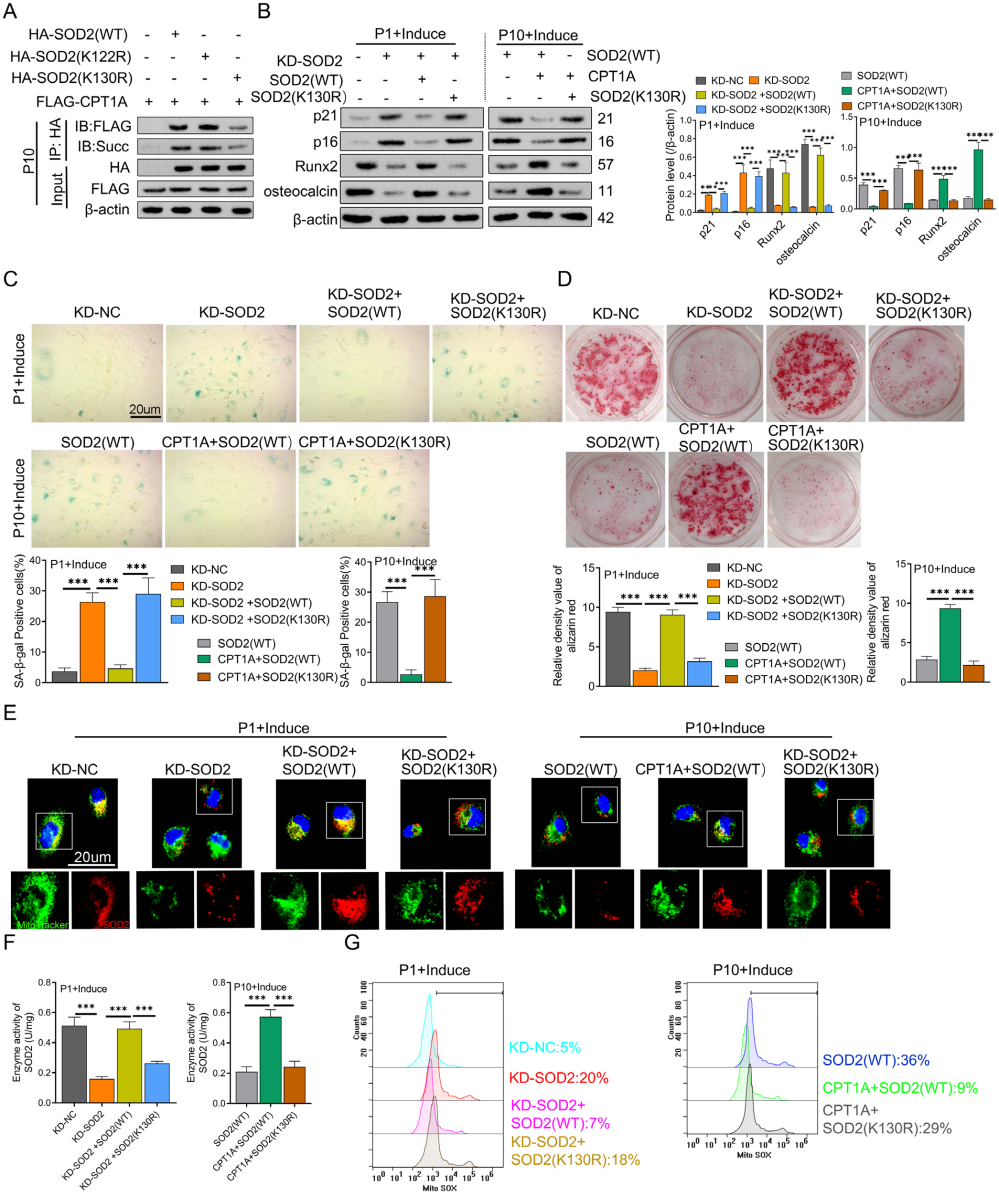
Verification of CPT1A's role in reducing senescence and restoring osteogenic differentiation in expanded BM‐MSCs by modifying SOD2 (K130) succinylation. (A) The combination of CPT1A and SOD2 succinylation after Co‐IP detection of SOD site mutation. (B) The expression levels of senescence‐related proteins p21 and p16 and osteogenic differentiation‐related proteins Runx2 and osteocalcin were detected by western blotting. (C) Beta‐galactosidase staining and quantification of the number of SA‐β‐gal‐positive cells. (D) Alizarin red staining was used to evaluate the osteogenic differentiation of cells. (E) Colocalisation images of SOD2 and mitochondria were obtained by immunofluorescence. (F) SOD activity detection kit was used to detect SOD activity. (G) Representative images of MitoSox in cells were analysed by flow cytometry to assess mitochondrial ROS levels. ***p < 0.001. *N* = 3, Statistical significance was determined by using one‐way ANOVA. *p* < 0.05 was considered significant.

This study showed the top 25 succinylation modification sites identified in the CPT1A (OE) group (Table 3), based on the results from mass spectrometry. The level of succinylation was upregulated at two (K130 and K122) of the three potential succinylation modification sites (K130, K122 and K68) of SOD2 (Table 4 and Figure S3). To verify whether K130 and K122 are succinylated, we constructed mutant plasmids, including K122R and K130R, and transfected them into P10 BM‐MSCs cells. Through Co‐IP experiments, we found that SOD2 (K122R) did not affect the binding with FLAG‐CPT1A or succinylation levels, whereas SOD2 (K130R) reduced the binding with FLAG‐CPT1A or succinylation levels. This indicates that the binding of CPT1A with SOD2 at K130 promotes the succinylation modification of SOD2 (Figure 6A). To verify that SOD2 (K130) is a modification site mediated by CPT1A for succinylation of SOD2, we knocked down endogenous SOD2 in P1 BM‐MSCs cells and then transfected with SOD2 (WT) or SOD2 (K130R) mutant plasmids. Similarly, after overexpressing CPT1A in P10 BM‐MSCs cells, we transfected these cells with SOD2 (WT) and SOD2 (K130R) mutant plasmids and performed the following experiments. First, Western Blot analysis showed that in Passage 1 BM‐MSCs, knocking down SOD2 significantly increased the protein expression of p21 and p16 and decreased the expression of Runx2 and osteocalcin. Transfection with SOD2 (WT) reversed this effect, whereas the expression levels of the SOD2 (K130R) mutant plasmid could not be reversed. In passage 10 BM‐MSCs, after overexpression of CPT1A and subsequent transfection with SOD2 (WT), compared to SOD2 (WT), the expression of p21 and p16 decreased and the expression of Runx2 and osteocalcin increased. However, when CPT1A was overexpressed and then SOD2 (K130R) was transfected, there were no significant changes in the expression levels of senescence and osteogenic differentiation‐related proteins (Figure 6B). This suggests that mutated CPT1A may affect the senescence phenotype and osteogenic differentiation capacity of BM‐MSCs cells by acting on the SOD2 (K130R) site.

**TABLE 3:** The top 25 succinylation modification sites were identified by mass spectrometry.

Protein accession	Gene name	Position	Amino acid	B/A ratio	B/A‐regulated type	PEP	Score	Charge	Modified sequence
P04179	SOD2	130	K	3.797792476	Up	1.7615E‐21	153.769628	2	DFGSFDK(1)FK
P04179	SOD2	122	K	3.463263186	Up	3.3104E‐21	162.070674	2	GELLEAIK(1)R
O14874	BCKDK	184	K	3.391515374	Up	0.034787056	96.69523586	2	ANVAK(1)AGLVDDFEK
P15531	NME1	100	K	3.209760174	Up	2.77734E‐13	169.2926137	2	VMLGETNPADSK(1)PGTIR
P55084	HADHB	188	K	3.011619694	Up	2.8979E‐05	157.0862356	2	LMLDLNK(1)AK
P25705	ATP5F1A	261	K	2.986304293	Up	0.021629647	115.0038297	2	STVAQLVK(1)R
Q9H845	ACAD9	239	K	2.857961759	Up	0.035082613	200.3661564	2	TEVVDSDGSVK(1)DK
P31146	CORO1A	132	K	2.817833109	Up	0.009845871	152.4233808	2	MYK(1)EEGLK
P23528	CFL1	19	K	2.728931286	Up	0.019341961	108.4265852	2	VFNDMK(1)VR
Q01518	CAP1	376	K	2.654919272	Up	0.040615141	195.7264176	2	TAK(1)LQDFK
P12694	BCKDHA	389	K	2.591462162	Up	0.048935876	189.941931	1	K(1)GEDFVK
P42765	ACAA2	270	K	2.42962924	Up	0.028063832	134.4430945	3	K(1)HNFTPLAR
P00441	SOD1	123	K	2.288758758	Up	1.29765E‐09	180.186749	2	TLVVHEK(1)ADDLGK
P19367	HK1	344	K	2.216573839	Up	0.024051878	131.4378202	2	FNTSDVSAIEK(1)NK
P30044	PRDX5	83	K	2.119090578	Up	4.30192E‐05	106.5460913	2	VNLAELFK(1)GK
P00505	GOT2	296	K	2.109452733	Up	4.59224E‐05	92.46041283	2	VGAFTMVCK(1)DADEAK
P41250	GARS1	224	K	2.093907116	Up	0.032414723	205.4540232	2	AHLQK(1)LMSDK
P30048	PRDX3	91	K	2.092675111	Up	4.58419E‐05	106.0415829	2	DLSLDDFK(1)GK
P40939	HADHA	531	K	2.054865886	Up	2.63871E‐05	215.7313942	2	DTSASAVAVGLK(1)QGK
P0DPI2	GATD3	261	K	2.042135473	Up	0.015060946	62.54926777	2	K(1)VLELTGK
P04075	ALDOA	13	K	2.000306348	Up	0.048947907	185.8658172	2	K(1)ALTSFER
P99999	CYCS	28	K	1.978949651	Up	0.019358047	50.19268082	2	ELK(1)EIQYGIR
P07954	FH	80	K	1.962952073	Up	0.042455393	95.0862736	2	STMNFK(1)IGGVTER
P50990	CCT8	459	K	1.838772417	Up	0.016920009	143.1975137	2	ALAENSGVK(1)ANEVISK
O95139	NDUFB6	66	K	1.788126822	Up	4.11425E‐13	186.9945211	2	MVHGVYK(1)K

**TABLE 4:** In the CPT1A (overexpression, OE) group, SOD2‐K succinylation was identified at three sites.

Protein accession	Position	Amino acid	B/A Ratio	Regulated Type	Gene name	Modified sequence	A1 (NC)	A2 (NC)	B1 (OE)	B2 (OE)
P04179	130	K	3.80	Up	SOD2	DFGSFDK(1) FK	0.38	0.43	1.67	1.445
P04179	122	K	3.46	Up	SOD2	GELLEAIK (1) R	0.46	0.399	1.580	1.39
P04179	68	K	0.92	None	SOD2	NVTEEK(1)YQEALAK	0.24	0.26	0.22	0.23

To further validate this hypothesis, we assessed the cells' senescence degree and osteogenic differentiation capacity. SA‐β‐gal‐staining results showed that, compared to the control group, knocking down SOD2 increased the number of senescent cells, which was reversed by transfection with SOD2 (WT), but not with the SOD2 (K130R) mutant plasmid (Figure 6C). Alizarin red‐staining results indicated that, compared to the control group, knocking down SOD2 reduced the intensity of alizarin red staining, which was reversed by transfection with SOD2 (WT), but not with the SOD2 (K130R) mutant plasmid (Figure 6D). Next, immunofluorescence (IF) assays revealed that, in P1 BM‐MSCs, knocking down SOD2 significantly reduced the colocalisation of SOD2 with mitochondria. This reduction was reversed by transfection with SOD2 (WT), whereas the colocalisation intensity remained similar in the group transfected with SOD2 (K130R). In P10 BM‐MSCs, after CPT1A overexpression, the colocalisation intensity of SOD2 with mitochondria increased in the group transfected with SOD2 (WT), but not in the group transfected with SOD2 (K130R) (Figure 6E).

To verify the effect of CPT1A‐mediated SOD2 (K130R) on SOD2 enzyme activity, SOD activity assays showed that in P1 BM‐MSCs, knocking down SOD2 significantly reduced SOD2 enzyme activity, which was reversed by transfection with SOD2 (WT), but not with SOD2 (K130R). Similarly, in P10 BM‐MSCs, overexpression of CPT1A followed by transfection with SOD2 (K130R) did not improve SOD2 enzyme activity, whereas transfection with SOD2 (WT) significantly improved it (Figure 6F). Finally, the mitochondrial mtROS assay results showed that knocking down SOD2 significantly increased the levels of reactive oxygen species (ROS) in mitochondria. Transfection with SOD2 (WT) reversed the increase in ROS levels, whereas transfection with SOD2 (K130R) failed to reverse this effect. Moreover, after overexpression of CPT1A, transfection with SOD2 (K130R) did not reduce ROS levels, whereas transfection with SOD2 (WT) significantly decreased ROS levels (Figure 6G). In summary, CPT1A improves the senescence and osteogenic differentiation of BM‐MSCs by promoting succinylation at the K130 site of SOD2, which enhances SOD2 enzyme activity and mitochondrial localisation.

## 4 Discussion

MSCs show great therapeutic potential in regenerative medicine and tissue engineering due to their self‐renewal ability and multidirectional differentiation potential. They can differentiate into a variety of cell types, including bone cells, cartilage cells and fat cells, to repair damaged tissue and have the ability to regulate the immune response. However, many studies have shown that MSCs will senescence during in vitro culture, and their osteogenic differentiation ability will gradually weaken. In the early stage of in vitro culture, MSCs would undergo a rapid proliferation period, but with the increase of passage times, the proliferation rate would gradually slow down. The morphology of MSCs changed, such as the cell size increasing and the shape becoming irregular. In addition, the pluripotency of MSCs decreased with the increase of culture time, which showed that the differentiation ability of osteogenesis, lipogenesis and chondrogenesis decreased [21, 22]. Therefore, an in‐depth exploration of the biological changes in the continuous passage of MSCs in vitro will help us develop new strategies to extend their useful life, improve treatment efficiency and ensure their safety in clinical applications.

Our study found that with the increase in the number of passages, the cellular senescent phenotype was enhanced and the osteogenic differentiation capacity was weakened, consistent with previous research findings. The accumulation of ROS during cell senescence is one of the key promoting factors. Excessive ROS can lead to oxidative stress, accelerating cell ageing and functional degeneration. Free fatty acids, such as palmitate, can induce endoplasmic reticulum stress and oxidative stress, leading to apoptosis of senescence‐associated β cells [23]. CPT1A can promote β‐oxidation of fatty acids, increasing the efficiency of mitochondrial energy production, which may help maintain the stability and integrity of the mitochondrial membrane, thereby reducing ROS production and mitigating oxidative stress [24]. Recently, the relationship between CPT1A and cell ageing has received increasing attention. Restoring CPT1A expression enhances mitochondrial fatty acid oxidation (FAO) in the kidneys, improves mitochondrial homeostasis and function, and thus helps reduce I/R injury and delay the ageing process [25]. The decrease in CPT1A expression promotes endothelial cell ageing, while its overexpression or improvement of fatty acid metabolism can slow down this process [26]. Our study also found that after multiple passages in vitro, the expression of CPT1A in BM‐MSCs was downregulated, the expression of senescence‐associated proteins p21 and p16 significantly increased, the number of β‐galactosidase‐positive cells increased and the expression of osteogenic differentiation‐related proteins Runx2 and osteocalcin decreased. This suggests that the downregulation of CPT1A after multiple passages of BM‐MSCs may accelerate ageing and weaken osteogenic differentiation capacity, consistent with previous research results. Additionally, in some tumour or osteoarthritis studies [26, 27], the downregulation of CPT1A has been shown to inhibit cell ageing, leading us to speculate that the role of CPT1A may vary in different cell types due to differences in the metabolic pathways involved, the cellular environment and the physiological or pathological pressures faced.

Mitochondria are the primary site for energy production within cells and a significant source of superoxide anions. SOD2 is an important antioxidant enzyme in the mitochondria and cytoplasm of cells. Enhancing its activity can effectively clear superoxide anions produced within the mitochondria, protecting the mitochondria from oxidative damage [28]. Studies have shown that the overexpression of SOD2 can protect mesenchymal stem cells in the brain and improve the recovery of neural inflammation and brain injury in traumatic brain injury mice [29]. Exosomes derived from human umbilical cord MSCs carrying the mitochondrial antioxidant enzyme Mn‐SOD can alleviate oxidative stress and play a protective role in liver ischaemia–reperfusion injury [25]. Despite the reported role of SOD2 in resisting oxidative damage in MSC therapy, whether SOD2 is involved in the ageing and osteogenic differentiation of MSCs regulated by CPT1A has not yet been studied. Our findings show that after osteogenic induction, the knockout of CPT1A in BMSCs led to an increase in the expression of senescence‐associated proteins p21 and P16, a significant increase in the number of β‐galactosidase‐positive cells and a decrease in the expression of osteogenic‐related proteins Runx2 and osteocalcin, with a reduction in calcium nodule formation. This indicates that inhibiting CPT1A promotes cell ageing and inhibits the osteogenic differentiation capacity of BMSCs. Simultaneously, the distribution of SOD2 in mitochondria was reduced, the enzymatic activity of SOD2 was significantly reduced and the level of mitochondrial ROS increased. The overexpression of CPT1A had the opposite effect on osteogenically induced BMSCs, while the protein expression of SOD did not change significantly. This suggests that CPT1A helps to promote the distribution of SOD2 in mitochondria, enhance its enzymatic activity and improve its antioxidant function, thereby slowing down the ageing process caused by mitochondrial dysfunction. To further determine the effect of CPT1A on the stability of the SOD2 protein, we treated BM‐MSCs with the protein synthesis inhibitor cycloheximide. The results showed that CPT1A did not affect the stability of the SOD2 protein. Our results demonstrate that CPT1A influences cell ageing and osteogenic differentiation by promoting the mitochondrial distribution of SOD2, enhancing its enzymatic activity and improving its antioxidant capacity.

Recent studies have shown that CPT1A plays a role in energy metabolism and is involved in posttranslational modification of proteins, particularly succinylation. Succinylation modification is ubiquitous in biological processes and can significantly change proteins' physicochemical properties and functions. Research has shown that CPT1A‐mediated succinylation modification plays an important role in various cancers. Specifically, CPT1A promotes the succinylation of S100A10, enhancing the invasive ability of human gastric cancer [11]. Additionally, CPT1A promotes the succinylation of MFF at the K302 site, which helps prevent Parkin‐mediated ubiquitin–proteasome degradation and significantly inhibits ovarian cancer progression [14]. Furthermore, CPT1A directly binds to SP5 and promotes its succinylation, which in turn enhances SP5's binding to the PDPK1 promoter, thereby increasing PDPK1 transcription and promoting prostate cancer progression [30]. Therefore, we speculate that CPT1A may influence the ageing and osteogenic differentiation of BMSCs by affecting the succinylation modification of SOD2. Experimental findings show that the increase in cell passages significantly reduces the overall succinylation level within the cells. Through Co‐IP experiments, we found that after knocking down CPT1A, the binding of SOD2 to CPT1A and the succinylation modification of SOD2 were reduced considerably, while this binding and modification were enhanced after the overexpression of CPT1A, and the mRNA expression level of SOD did not change significantly.

Additionally, we used the CPT1A inhibitor ST1326 to validate these findings again, and the results showed that ST1326 could inhibit the activity of CPT1A, affect the succinylation process of SOD2 and inhibit the entry of SOD2 from the cytoplasm into mitochondria, thus reducing the antioxidant function and enzymatic activity of SOD2. Furthermore, we verified through rescue experiments that the K130 site of SOD2 is the modification site for CPT1A‐mediated succinylation. The discovery of CPT1A‐mediated SOD2 succinylation modification provides a new perspective for understanding the molecular mechanisms of BMSC ageing and osteogenic differentiation.

Although this study has made some progress in exploring the scientific application of BM‐MSCs in medical repair and regenerative medicine by alleviating senescence during culture, there are still some limitations. Firstly, maintaining the stability of BM‐MSCs after the large‐scale expansion in vitro is a potential problem that needs further study. Secondly, this study mainly focuses on in vitro experiments, lacking in vivo experiments. The research team is currently establishing a conditional gene knockout mouse model for in vivo experimental studies. We will submit and publish these findings in real time to further validate and expand the current research results.

**FIGURE 7:**
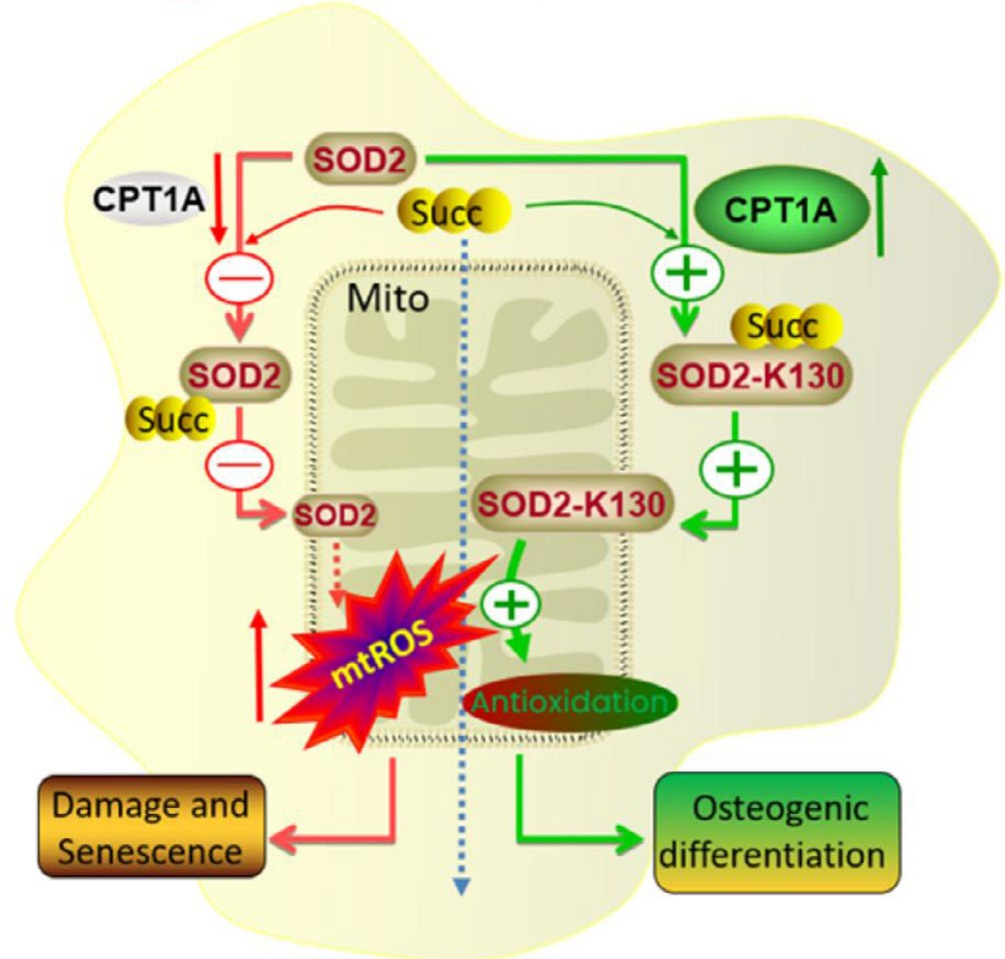
Hypothetical model. CPT1A promotes the succinylation of the SOD2 protein and enhances its accumulation and enzyme activity in BM‐MSCs, thereby alleviating cell ageing and promoting osteogenic differentiation.

In summary, this study revealed that CPT1A plays a key role in alleviating BM‐MSC senescence and promoting osteogenic differentiation by promoting SOD2 (K130) site succinylation and enhancing SOD2 accumulation and enzyme activity in mitochondria (Figure 7). This finding provides a new idea for improving the culture conditions of MSCs and enhancing their osteogenic differentiation ability, which is of great significance for optimising the application of MSCs in regenerative medicine.

## Author Contributions

Experiments: Xin Hua Yin, Xiao Yuan Wang, Shi Chang Liu; Study concept and design: Xu Xu Chen, Liang Yan, Liang Li, Gao Le He; data acquisition: Xin Hua Yin, Xiao Yuan Wang; data analysis and interpretation: Shi Chang Liu, Xu Xu Chen, Ming Yang; drafting of the manuscript: Xin Hua Yin, Xiao Yuan Wang; statistical analysis: Liang Yan, Liang Li, Gao Le He, Ming Yang; study supervision: Zhong Kai Liu.

## Conflicts of Interest

The authors declare no conflicts of interest.

## Supporting information


**Figure S1.** BM‐MSCs cell identification. (A) The cell surface markers CD34, CD73, CD90 and CD105 of MSC were detected by flow cytometry; (B) Alizarin red staining for osteocytes; (C) Oil red O staining for adipocytes; and (D) Alcian blue staining for chondrocytes. Magnification, ×200. Scale bar = 50 μm.

**Figure S2.** Silver staining and mass spectrometry of CPT1A‐associated proteins. (The arrow shows the location of SOD2).

**Figure S3.** Secondary mass spectrometry of protein identified by SOD2‐K succinylation.

## Data Availability

The data in this study can be obtained from the corresponding author.
