# Supplementary figures and images for: CPT1A Alleviates Senescence and Restores Osteogenic Differentiation of BM‐MSC Through SOD2 Succinylation
Source: J Cell Mol Med. 2025 Mar 11;29(5):e70473. doi: 10.1111/jcmm.70473 (PMC11897055; doi:10.1111/jcmm.70473)

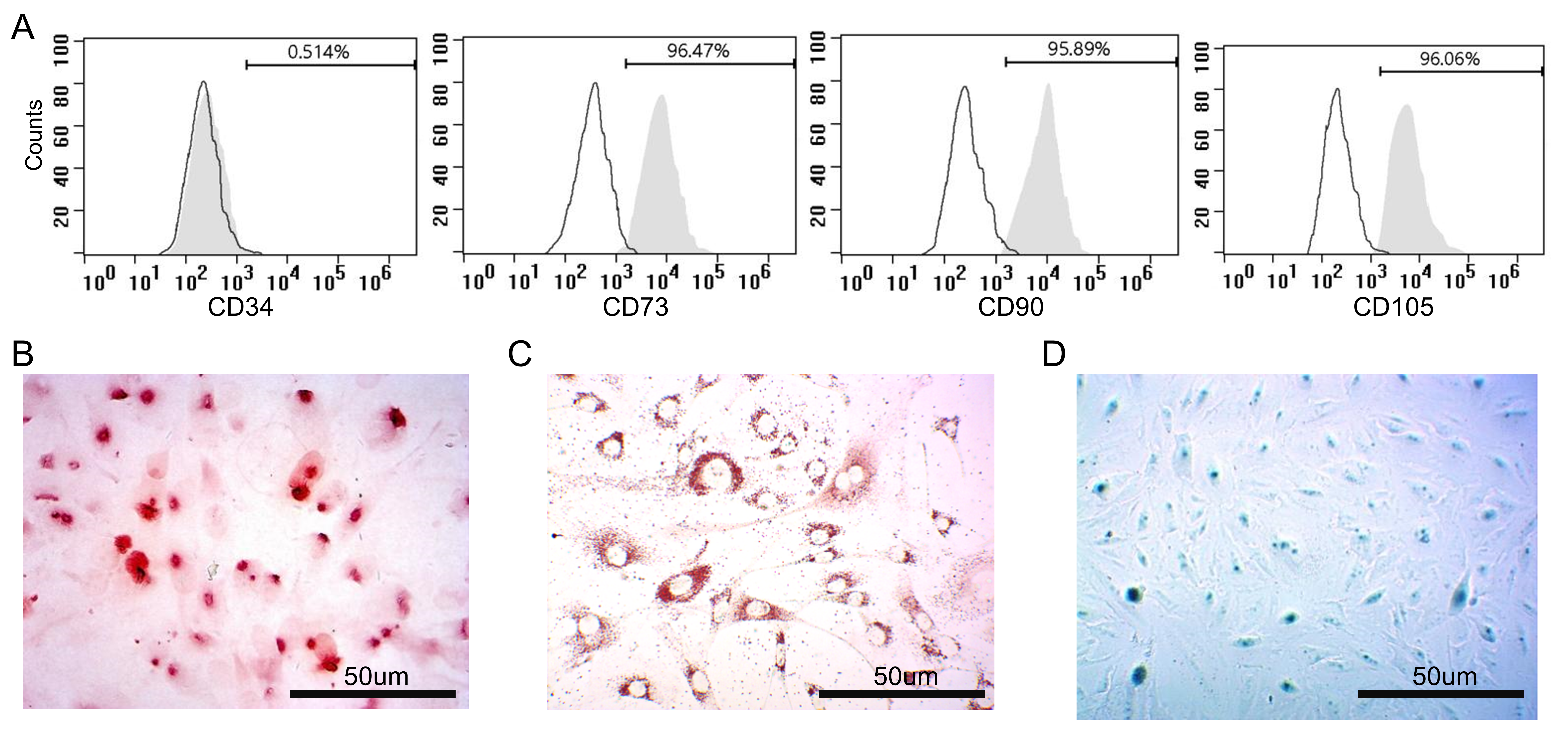

Supplement: Supplementary file 1 — Figure S1. BM‐MSCs cell identification. (A) The cell surface markers CD34, CD73, CD90 and CD105 of MSC were detected by flow cytometry; (B) Alizarin red staining for osteocytes; (C) Oil red O staining for adipocytes; and (D) Alcian blue staining for chondrocytes. Magnification, ×200. Scale bar = 50 μm. [file JCMM-29-e70473-s003.tif]

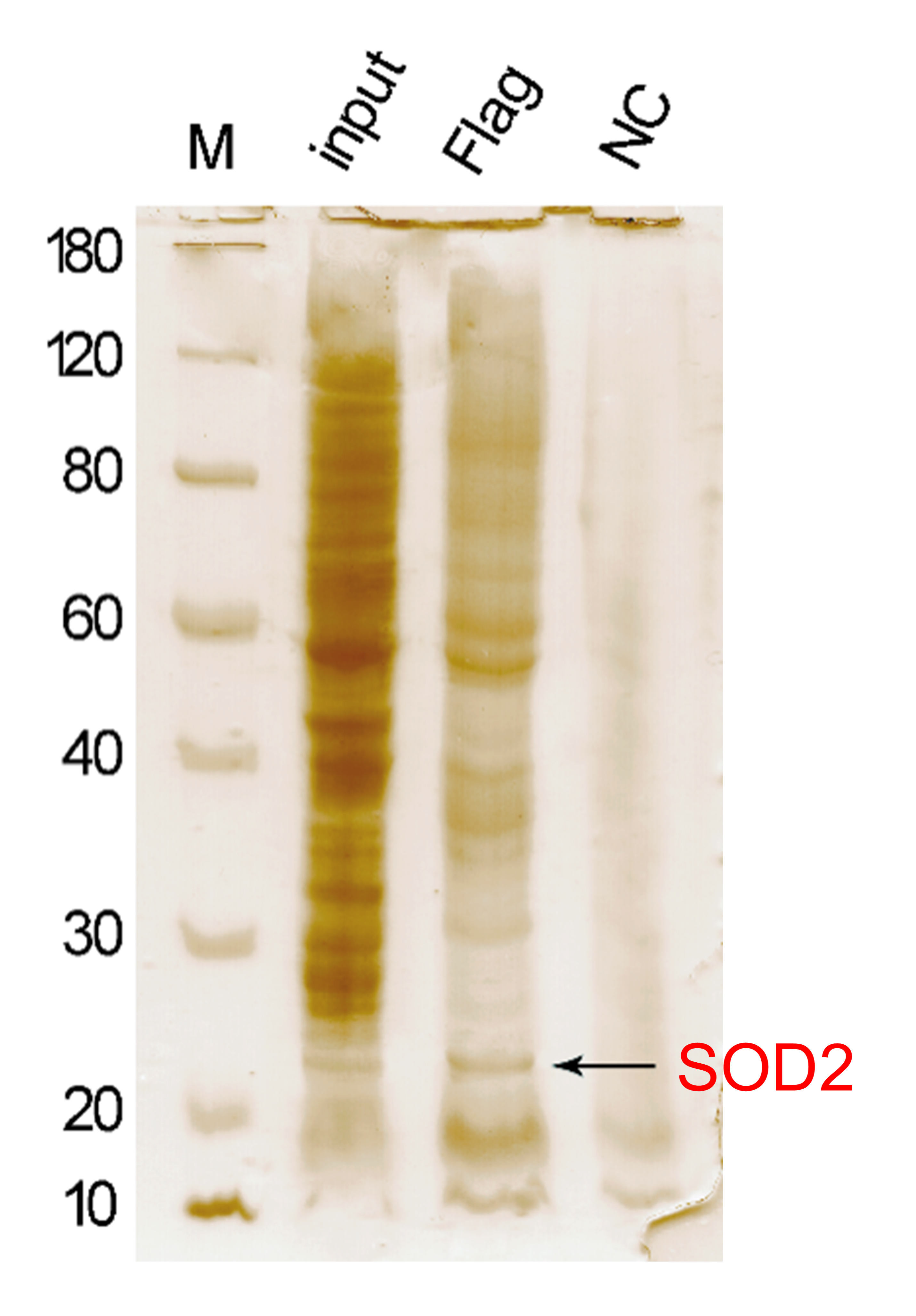

Supplement: Supplementary file 2 — Figure S2. Silver staining and mass spectrometry of CPT1A‐associated proteins. (The arrow shows the location of SOD2). [file JCMM-29-e70473-s001.tif]

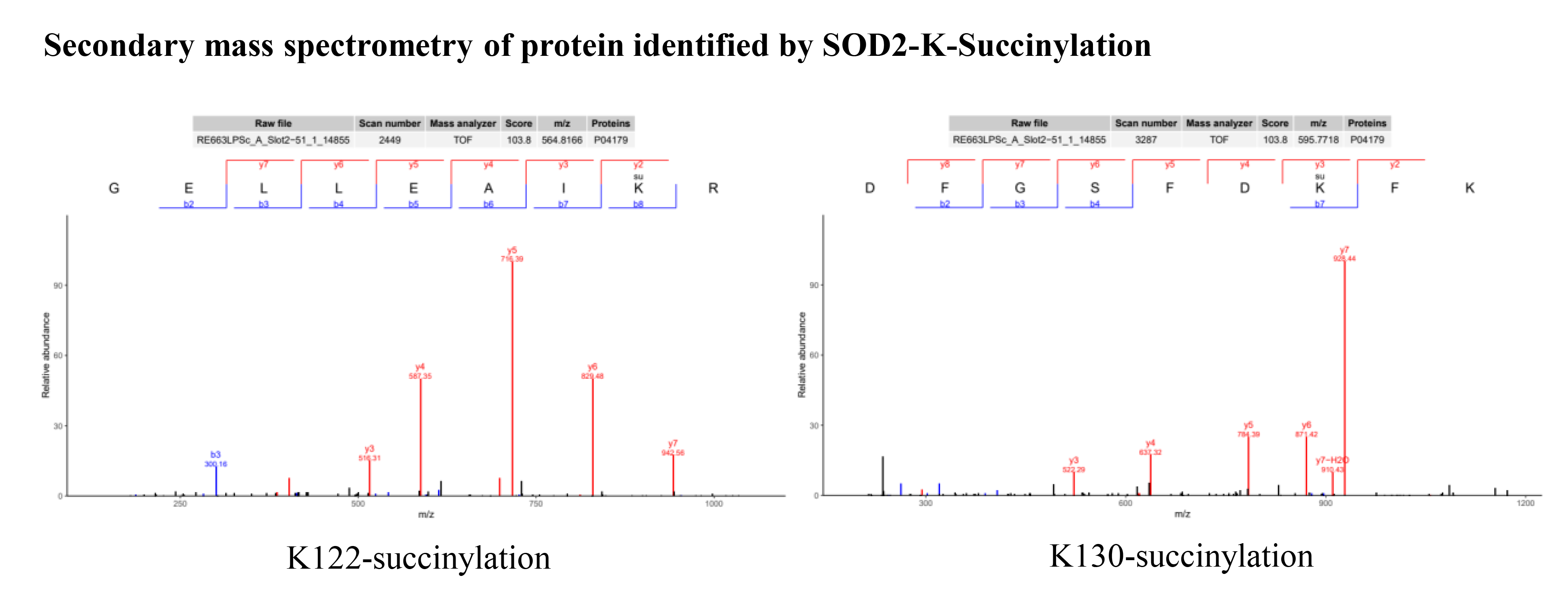

Supplement: Supplementary file 3 — Figure S3. Secondary mass spectrometry of protein identified by SOD2‐K succinylation. [file JCMM-29-e70473-s002.tif]
